# Don’t Look Up! Individual Income Comparisons and Subjective Well-Being of Students in Thailand

**DOI:** 10.1007/s10902-022-00604-4

**Published:** 2022-12-01

**Authors:** Thomas Dufhues, Judith Möllers, Antje Jantsch, Gertrud Buchenrieder, Laura Camfield

**Affiliations:** 1 External Environment for Agriculture and Policy Analysi, Leibniz Institute of Agricultural Development in Transition Economies (IAMO), Halle (Saale), Germany; 2 Institute of Sociology and Economics, Universität der Bundeswehr München, Munich, Germany; 3 School of International Development (DEV), University of East Anglia, Norwich, UK

**Keywords:** Subjective well-being, Income comparisons, Reference groups

## Abstract

Empirical evidence supports the hypothesis that an individual’s position in an income stratum—more than the absolute income level—determines subjective well-being. However, studies on subjective well-being suffer from a critical methodological weakness: they use exogenously defined reference groups. Our study addresses this point by applying an innovative new survey instrument. We ask respondents to identify individual reference persons for income comparisons. We find that these reference persons come from a range of social groups. Interactions between personality traits and the direction of income comparisons lead to different levels of subjective well-being. This highlights the importance of collecting information on personality traits in research on subjective well-being. We conclude that questions about self-defined individual income comparisons can be a valuable and straightforward addition to future surveys.

## Introduction

As early as 1974, Richard Easterlin was one of the first economists to link happiness data to income, using data from surveys conducted by the Gallup Poll and the American National Opinion Research Centre. He compared the self-reported happiness of U.S. citizens. On the micro-level, he found a positive association of happiness and income. On the macro-level and contrary to expectations, he found no relationship between their reported happiness and average income over time, even though income had increased substantially. This became known as the ‘Easterlin Paradox' (Easterlin, 1974: 116). In 1995, he reaffirmed this result “with somewhat greater assurance than twenty years ago” (Easterlin, 1995: 35).^1^ The Easterlin paradox is mainly explained by social comparisons: the positive effect of one person’s income growth is offset by the negative effect of the reference group’s income growth (Clark et al., 2008: 99). Subjective well-being is, therefore, not only determined by absolute income levels, but also by the relative position within an income stratum (Hopkins, 2008: 351). Following these ground-breaking ideas, a large body of literature has been devoted to the effect of relative income positions on subjective well-being (Clark et al., 2010: 407) often described as the economics of happiness.

Social comparisons that trigger income-related changes in subjective well-being have usually been captured through income reference groups. The notion that individuals compare themselves within reference groups is recognised, for example, in the theoretical concepts of relative deprivation, relative status, and the social frame of reference (Perez-Asenjo, 2011: 1413). Despite the importance assigned to these concepts and some understanding that people often compare themselves to similar others (family, friends, work colleagues), evidence of how individuals delineate their reference group for income comparison is lacking (Clark & Senik, 2010: 573; Clark et al., 2013: 1; Gugushvili, 2020: 3; Senik, 2009: 409).

Instead of delineating individualised reference groups, most empirical studies settle for exogenously predefined collectives for comparisons, such as the citizens of a country, region, or village, or people in the same social space, such as colleagues, or with the same socio-demographic characteristics, such as gender (Ferrer-i-Carbonell, 2005: 1001; van Praag, 2011: 117). These predefined reference groups are, however, debatable. First, most individuals are likely unaware of the reference income assumed by the scholar for the comparison. This could be, for example, the average income of people living in a certain region.^2^ Assuming that people compare themselves to an abstract reference group about which they lack knowledge seems unlikely. Second, even if people were aware of such income figures, it is difficult to determine the direction of the comparison. The relationship between income comparison and subjective well-being is likely linked to whether people compare themselves upward or downward. Predefined reference groups fail to provide clear insights in this regard. Third, Diener and Fujita (1997: 330) show that people actively select whom they compare to, indicating the existence of a distinct reference group rather than a social collective (Bellani, 2012: 496, 499).

Irrespective of the direction, social comparisons affect people’s subjective well-being (Clark et al., 2008: 99). These effects, in turn, can both enhance and reduce subjective well-being (cf. Smith, 2000: 175 for a comprehensive literature overview). The lack of scholarly consensus on whether reference incomes positively or negatively affect an individual’s subjective well-being may be an artefact of a priori and externally defined reference groups (Brown et al., 2015: 47). An a priori and external determination of reference groups makes it impossible to determine the characteristics of an individual’s actual reference group (van Praag, 2011: 117) and whether it represents an individual’s actual reference group or not (Clark et al., 2009: 519–520; Wu, 2020: 3). A few exceptions exist where studies allowed respondents to (partly) define their reference groups according to role relationship by choosing from a list that included family, friends, colleagues, and neighbours (Clark & Senik, 2010: 591; Goerke & Pannenberg, 2015: 96; Gugushvili, 2020: 9; Hyll & Schneider, 2014: 334; Knight et al., 2009: 637). However, with one exception, these studies did not permit multiple list items to be combined. Furthermore, only Goerke and Pannenberg (2015: 96) explicitly looked at the direction of income comparison by asking for the income differential between the respondents and their selected reference group. While these studies are doubtless a step in the right direction, they do not reveal the properties of the “true” reference group and largely still work within predefined parameters. There is also a vast body of social-psychological research, which provides valuable insights into social comparisons in general. This literature is summarised prominently in Buunk and Gibbons (2007).

The problems with the a priori definition of reference groups call for new and innovative survey instruments that capture individuals’ true reference groups (Perez-Asenjo, 2011: 1438). Therefore, this paper aims to identify individualised reference groups empirically. We address the following research questions: (1) Who are the “true” reference individuals with whom a person compares their income? Answers to this question can provide clues as to whether these reference individuals are similar to the commonly used a priori defined reference groups. In addition, we can discuss comparison patterns based on a fine-tuned distinction of upward and downward comparisons. In the next step, we ask: (2) How are individual comparison patterns linked to subjective well-being? The answers to these questions contribute to closing a significant gap in research regarding the composition and characteristics of individual reference groups, directions of comparisons (e.g. upward or downward), and their relationship to subjective well-being.

Our analysis and econometric model are theoretically rooted in the literature on well-being and social comparisons. To identify reference individuals, we apply an innovative self-report method based on the name generator, a survey instrument that originated in sociology. The resulting data allows us to identify individual comparison patterns (e.g. upward or downward) and discuss the characteristics of different types of comparers. The novel instrument was applied for the first time to students at two universities in Thailand in 2019. Our sample consists of 276 complete interviews. We use regression analysis to determine whether the different comparison patterns explain subjective well-being.

## Literature Review

### A Social Comparison Framework

**Fig. 1:**
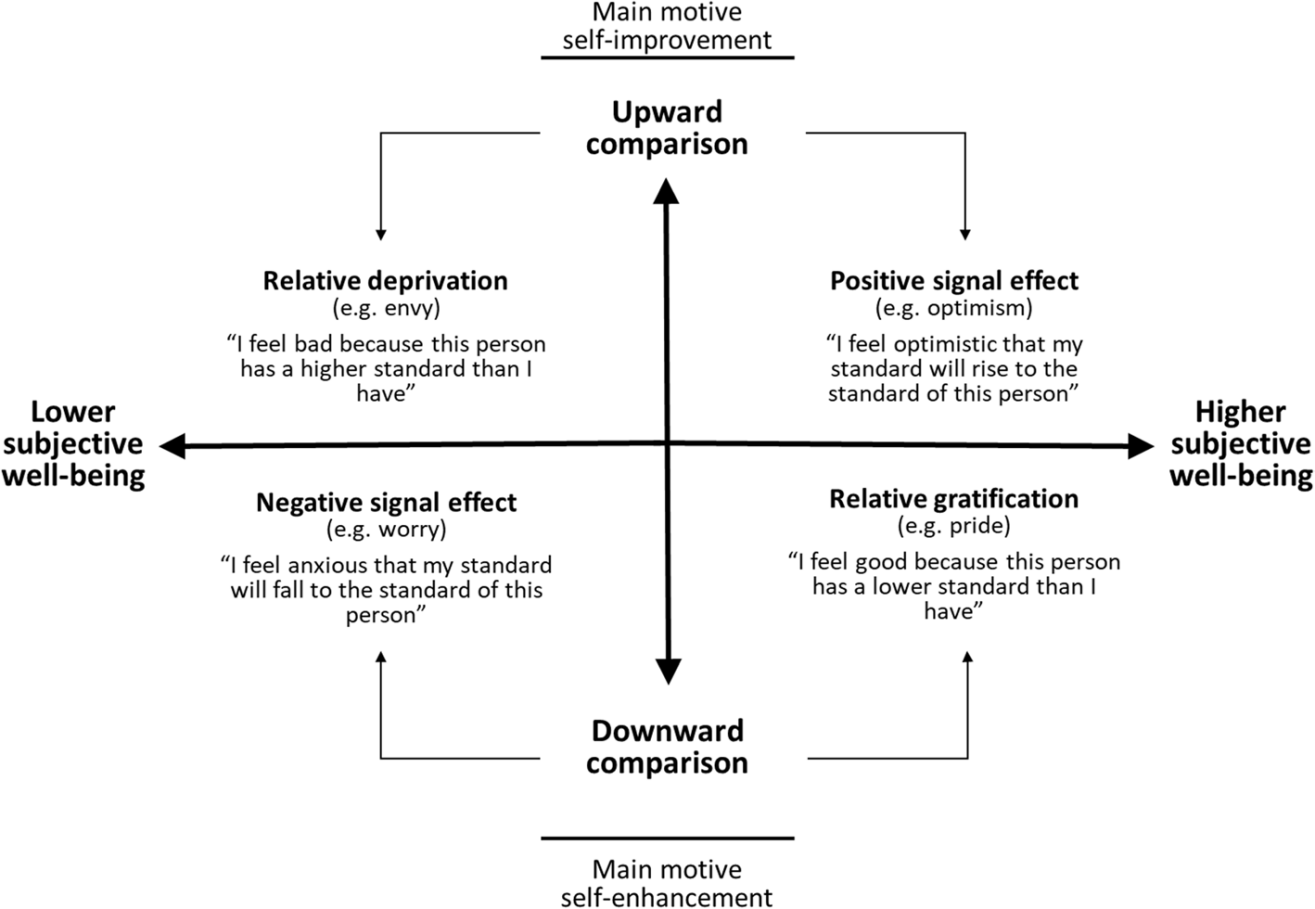
Framework for the identification of individualised reference groups – social comparisons and their effect on subjective well-being. Source: Own figure inspired by Smith (2000: 176)

According to the literature, people have two main motives when choosing a reference group for comparison: self-improvement and self-enhancement (Falk & Knell, 2004, 420–421; Wood & Taylor, 1991: 28) (see Fig. 1). Self-improvement focuses on “getting better” and is future-oriented. Self-enhancement centres on “feeling better” and is presence-oriented (Taylor et al., 1995: 1278). When the motive of comparison is self-improvement, people tend to compare themselves to individuals who are better off. This may motivate them and cause them to perform better (Wood & Taylor, 1991: 27). Positive outcomes of upward comparisons are likely to occur whenever there are prospects for individual development (Senik, 2004: 2101), and upward comparisons can, for example, trigger an increase in the perception of self-efficacy (Major et al., 1991: 254). People may also feel optimistic about their future income based on their reference group’s income. This is positively related to subjective well-being, as individuals expect their income to rise over time. This positive signal effect is referred to as the *tunnel effect* (Akay et al., 2012: 421; Hirschman & Rothschild, 1973: 545). However, upward comparisons can also lead to feelings of envy that may decrease an individual’s subjective well-being (Brockmann & Yan, 2013: 142; Taylor et al., 1983: 27). This effect is called the *relative deprivation effect,* which arises when people compare themselves to others who have achieved something attainable and desirable that they themselves have not yet achieved (Runciman, 1966: 9).^3^

The motive of self-enhancement is just as important. Here, people often make themselves feel better by comparing themselves with those they consider worse off. In this case, downward comparisons may increase subjective well-being through tension reduction and feelings such as pride (Hopkins, 2008: 368; Wills, 1981: 245, 265). This is known as the *relative gratification effect* (see, e.g. Jantsch et al., 2022: 12). However, this will only happen if the respondent feels safe from experiencing a similar fate. If this is not the case, negative feelings may develop, such as worry and fear of social decline, and subjective well-being decreases (Lockwood, 2002: 355). This can then be interpreted as a negative signal effect, which is the counterpart to the above-mentioned tunnel effect.

In short, upward comparisons aim at self-improvement, while downward comparisons aim at self-enhancement (Taylor et al., 1995: 1283). However, the net effects may be negative, zero or positive depending on the emotional consequences involved: upward comparisons can be inspiring or demoralising, while downward comparisons can be elevating or depressing (Fujita, 2008: 254).

### Self-Reported Income Reference Groups

The methods used to measure social comparisons and identify reference individuals within the various social sciences can be split into three groups. The first group comprises selection methods in which participants choose comparison targets from a list defined by the researcher. This approach usually takes place in a laboratory. The second group is made up of reaction methods in which participants are exposed to different comparison targets, and their responses are analysed. These also take place in a laboratory. The last group contains narrative methods, which rely on free responses based on qualitative interviews and self-reports such as self-recorded social comparison diaries^4^ or global self-reports (Gerber et al., 2018: 180). Global self-reports are especially suitable for quantitative research. They are simple to administer and, therefore, inexpensive. They can also be easily standardised and integrated into structured surveys. Researchers can directly ask respondents whom they compare themselves to, making it possible to identify individual reference persons, for example, for income comparisons.

However, many studies rely on exogenously defined reference groups. It is unclear if respondents actually use these groups for comparison or are aware of the income levels within them. In cases where respondents are allowed to define their reference groups, they can often only choose from a list of predefined groups, such as colleagues, schoolmates, or friends (selection method). Empirical evidence suggests that, in Europe, colleagues are the most important reference group (Clark & Senik, 2010: 576), and the relationship between subjective well-being and the incomes of others is stronger for colleagues than for family or friends (Clark et al., 2013: 16).^5^ Competitiveness, such as in a working environment, could reinforce the effect of social comparisons on subjective well-being, which may explain the importance of colleagues, as salaries are seen as a reflection of performance (Wolbring et al., 2013: 89, 98).

Most people have a limited range of individuals they compare to (Bellani, 2012: 496, 499). Research has shown that routine social comparisons of various kinds are made with the same individual(s) regardless of the topic (Corcoran & Mussweiler, 2009: 947; Mussweiler & Rüter, 2003: 467). As Festinger (1954: 120) postulated and McBride (2010: 263), for example, has empirically shown, comparisons are usually made with individuals with similar socio-demographic characteristics.^6^

Comparisons depend to a certain degree on the people an individual is exposed to. This is called “forced comparisons”. Here, reference individuals are mostly those who live close by or with whom close interaction exists, such as at work (Diener & Fujita, 1997: 330). For instance, Knight et al., (2009: 637) report that respondents in rural China considered people living in their village as their main reference group. Comparisons with people in an individual’s immediate environment often have a greater effect than comparisons with people in general. This is known as the “local dominance effect” (Zell & Alicke, 2010: 368), but it is not always the case, see. e.g. Becchetti et al., (2013: 187) or Bruchmann and Evans (2013: 427). However, reference individuals for social comparisons can also be people of no direct social relation, such as opinion leaders or celebrities (Hyman & Singer, 1968: 9). In general, as stated by Diener and Fujita (1997: 330), people have a much more active role in selecting reference persons than is suggested by “forced comparisons”.

Very few studies on subjective well-being look at the upward or downward direction of self-determined income comparisons. To the best of our knowledge, only one article discusses the direction of income comparisons based on reference groups determined using a selection method—Goerke and Pannenberg (2015: 95). They find that work-related income comparisons are mostly upward and lead to lower subjective well-being. Other researchers also suggest that income comparisons are mostly upward (Clark & Senik, 2010: 591; Ferrer-i-Carbonell, 2005: 1015). However, they draw this conclusion indirectly from the fact that the observed comparisons are negatively related to subjective well-being and, therefore, more likely to have been upward.

## Empirical Strategy

### Identification of Reference Individuals

We used a self-report method to identify an individual’s true reference persons and answer the first research question. This method is based on the *name generator*, a survey instrument that originated in sociology. Name generators are usually used to reveal the members of a respondent’s personal network by collecting information about certain domains of this network (McCallister & Fischer, 1978: 131). Respondents, for example, may provide a name to answer a question such as “Who can help you repair your car?” This name is then recorded as part of their personal network. Since we wanted to identify reference individuals used for income comparisons, we slightly modified the name generator and phrased it as follows:

We now ask you to consider which particular persons you are thinking of when comparing your income situation. These people may or may not be important in your life. You may not even know them personally, such as public figures. But they must be a point of reference. Please take a moment to mention (in anonymous form) one to max. six real people to whom you compare your income situation most likely/most frequently.^7^

Only a few researchers have applied a similar survey instrument in their studies to capture reference individuals (e.g. Kim et al., 2018: 521). However, respondents were asked to name only one reference individual in these studies. In other studies, respondents were asked to name several people to sample a network of their social contacts. It was then assumed that this network of social contacts was also their reference group (e.g. Olivos et al., 2021: 372). However, there is a difference between reference groups and personal networks: while members of a personal network are often aware that they are part of this network, members of a reference group usually are not. This is because people do not necessarily reveal to whom they compare themselves (Wills, 1981: 265).

After respondents named reference individuals, they were asked to provide more details about them, including their gender and the role relationship (e.g., friend/relative). This part of the survey is called the *name interpreter* (Snijders, 1999: 29). The literature recommends keeping self-administered online surveys short to prevent respondents from dropping out due to interview fatigue (Lang, 2007: 71). The number of questions to be answered in the name interpreter is multiplied by the number of individuals mentioned. Thus, limiting the number of names when applying name generators is advisable. Therefore, most name generator instruments restrict the number of possible answers in the lower single digits (see, e.g. Dufhues et al., 2011: 1206). Following pre-testing, we limited the number of reference individuals to six.

Past studies on social networks have shown that survey respondents can report on many network characteristics with reasonable accuracy (Marsden, 1990: 456). We assume this holds for reference individuals, too, although accuracy is less critical here. Instead, subjective perception of the characteristics of reference individuals is decisive for the comparison. Since comparisons are made with perceived characteristics of reference individuals rather than objective data, any misperception is intrinsically part of the subjective measurement and not an error (Inglehart et al., 2008: 279; Merton, 1995: 380). In the words of Clark et al., (2013: 16), “It is not what others actually do earn that matters, but rather what individuals believe they earn.”

When people are directly asked to whom they compare their income, certain biases may lead them to underreport reference individuals. Some respondents may not be aware that they compare themselves to others, i.e., *lack of awareness bias* (Buunk & Gibbons, 2006: 16) or forget the social comparisons they have made, i.e., *recall bias* (Gerber et al., 2018: 180). It is also possible that specific directions of social comparisons, such as downward comparisons (Wills, 1981: 246, 265), especially related to income, are frowned upon (Burchell & Yagil, 1997: 746). This can cause respondents to deliberately underreport them, i.e., *social desirability bias.*^8^ We used warm-up questions to reduce the lack of awareness, recall, and social desirability biases. We asked questions about social and income comparisons, including the social comparison orientation scale (SCO) of Gibbons and Buunk (1999: 145) and reference groups.

### The Econometric Model

We employed regression analysis to answer the second research question: How are different individual comparison patterns linked to subjective well-being? Our dependent variable, subjective well-being, was assessed through a single item question about life satisfaction: All things considered, how satisfied are you with your life?^9^ Respondents were allowed to select integer numbers in a range from one (1) to ten (10), similar to the famous Cantril ladder (Diener, 1984: 546; Powdthavee, 2009: 228). The numbers range from completely dissatisfied (1) to completely satisfied (10) with life as a whole. Ferrer-i-Carbonell and Frijters (2004: 641) state in their frequently cited paper that “assuming ordinality or cardinality of subjective well-being scores makes little difference”.^10^ We, therefore, treat our dependent variable as cardinal and perform OLS regressions. Thus, to explain the subjective well-being (*SWB*) of individual *i*, we use the following regression equation:

$$ SWB_{i} = \alpha CP_{i} + {\mathbf{BigFive}}_{i}^{{\prime}} {{\boldsymbol{\upbeta}}} + {\mathbf{SCO}}_{{\varvec{i}}}^{{{\prime}}} {{\boldsymbol{\upgamma}}} + {\mathbf{x}}_{{\boldsymbol{i}}}^{{{\prime}}} {{\varvec{\updelta}}} + \varepsilon_{i} , $$

where *CP* denotes the comparison pattern of individual *i*. We asked individuals about the income level of each of their reference individuals and whether it was lower, higher or similar. This allows us to capture the direction of the direct income comparisons, i.e. whether a respondent predominantly compares themselves to individuals with higher incomes (upward comparison), lower incomes (downward comparison), or the same level of income (horizontal comparison). We then aggregated the responses into one variable that defines the respondent’s comparison pattern. We created a set of four mutually exclusive categories of income comparison patterns: (i) mostly upward comparer, (ii) mostly horizontal comparer, (iii) no clear pattern of comparison (fuzzy comparer), and (iv) non-comparer. If at least two-thirds of the reference individuals had a higher (the same) income compared to the respondent, the respondent’s comparison pattern was defined as “mostly upward comparer” (52%) (“mostly horizontal comparer” (23%)). The group of respondents who compared themselves mostly downward was very small (only 3.6%). When we ran a regression with downward and horizontal comparers as separate categories, the signs of the dummy coefficients and the magnitude of the “comparison pattern” variable were similar, so we merged these groups. When neither pattern was predominant, we labelled the respondent’s comparison pattern as “fuzzy comparer” (14%). Finally, respondents who indicated that they did not compare their income with others were labelled “non-comparer” (11%).

An individual’s personality is a strong and consistent predictor of subjective well-being (Boyce et al., 2013: 287). To control for personality traits, we included measures of personality based on the standard Big Five (Goldberg, 1993: 26) and social comparison orientation, *SCO* (Gibbons & Buunk, 1999: 145). The vector BigFive contains variables on five personality dimensions: openness to experiences, extraversion, conscientiousness, agreeableness, and neuroticism. We measured these dimensions using a short scale developed by Rammstedt and John (2007: 210) (see Table A 1 in the appendix for the wording. The single personality traits were measured each with two single items; then the average of these two items was taken; the higher the score, the higher the respondents were placed on the personality trait).

The vector SCO contains two variables on other personality traits that are important for social comparisons (Hemphill & Lehman, 1991: 390): the “performance orientation” of social comparisons (*How am I doing compared to others?*) and the “opinion orientation” (*What do others think*/*How do others act in a similar situation to me?*)*.* Performance orientation may reflect the respondent’s status, as it measures how the respondents evaluate themselves compared to others. The opinion orientation covers more information-gathering, problem-solving and goal-oriented aspects of social comparisons. With these personality traits, we captured individual differences in the intensity of making social comparisons (Gibbons & Buunk, 1999: 145). We applied the shorter version of this scale developed by Schneider and Schupp (2014: 770) (again, see Table A 1 in the appendix for the wording. The performance orientation and opinion orientation were measured with three single items each; then the average of these three items was taken; the higher the score, the more respondents tended to compare socially).

The vector ${\varvec{x}}$ contains other control variables for socio-demographic and socio-economic characteristics, including the respondent’s net income, the amount of time they spend on social media per day, their gender, university location, and age. *α, β, γ* and *δ* represent parameters to be estimated, and *ε* is the idiosyncratic error term.^11^

### Research Region and Data

We chose to apply our novel survey instrument in Thailand. The reasons for this are twofold: First, recent research suggests that relative considerations concerning income also play a role in subjective well-being in developing countries, such as Thailand (Asadullah & Chaudhury, 2012: 949; Kingdon & Knight, 2007: 86; White & Lehman, 2005). Second, Thai society is highly collectivist (Hofstede, 2001: 176, 215). People tend to pick up signals on appropriate behaviour from groups (Begley & Tan, 2001: 549) and compare themselves to others more frequently—especially upwardly (Chung & Mallery, 1999: 340; White & Lehman, 2005: 232). Thus, Thailand is well suited for investigating individual reference groups.

In 2011, Thailand was categorised by the World Bank as an "upper middle-income country". Gross domestic product (GDP) per capita grew by an average of 3.6% per year from 2010 to 2019, reaching US$7,187. In 2020, the first year of the COVID-19 pandemic, GDP decreased by 6.7%. Although nearly one-third of the population still works in the agricultural sector, it contributes only 8.6% of GDP (World Bank, 2021). While absolute poverty has decreased remarkably, inequality increased over recent decades. Among the East and Southeast Asian countries, Thailand has one of the highest levels of within-country income inequality and is the fourth most unequal country in the world (Draper & Selway, 2019: 275; Laovakul et al., 2016: 40; Pasuk, 2016: 405; Warr, 2007: 138).

The survey instrument was developed using pre-testing, group discussions and qualitative interviews with a smaller number of students. Cognitive debriefing techniques were applied to better understand how people identify their reference individuals and how they compare themselves to them. A critical outcome of this pre-survey phase was the understanding that some guidance was needed to help respondents reflect on the topic before identifying their reference individuals. Warm-up questions were thus added to the questionnaire to ensure that this reflection took place.

We applied our survey instrument to a student sample. Students provide a very good research base in this context, as studying is very competitive, and people facing higher competition are more likely to compare themselves with others (Schneider & Schupp, 2014: 771; Wolbring et al., 2013: 89, 98). The survey took place in November and December 2019, before the COVID-19 pandemic started. Our Thai research partners facilitated access to students in two contrasting university locations: Chiang Mai University in Northern Thailand and Surindra University in Northeast Thailand. Chiang Mai University is a high-ranking university in a cosmopolitan and wealthy area. Surindra University is a smaller, lower-ranked provincial university in the relatively poor Isaan region. During their lectures on Mass Communication at Chiang Mai University and Economics and Primary Education at Surindra University, students were asked to participate in the survey. Students immediately answered the survey in class. The anonymised online survey was self-administered and took, on average, 15 min to complete. Although participation was voluntary. To our best knowledge, all students in the lecture room started to fill in the questionnaire. A dropout rate of about one-third indicates that students did not feel pressured to finish the survey. The dropouts may suggest a self-selection bias which is discussed in the limitation section below.

The sample comprises 276 valid student interviews. Seven interviews were excluded due to missing values.^12^ The sample is mostly made up of undergraduate students aged between 19 and 22 (90%). Like most Thai students, our respondents spend a lot of time on social media (30% spend more than seven hours per day on social media). About 70% of our respondents are female. This is mainly due to the two main study programmes of most of our respondents, Mass Communication and Primary Education, which are predominantly taken up by female students.

### Limitations of the Survey

Although Cantril-like single-item questions to measure subjective well-being are a well-proven and frequently used survey instrument, they are less robust than multi-item instruments such as the Satisfaction-with-Life-Scale (SWLS) developed by Diener et al. (1985). We opted for Cantril-like single-item questions to avoid fatigue among the respondents during the online survey. Moreover, order effects in the survey, such as the sequence of questions in general, the range of warm-up questions before our survey instrument, and the position of the subjective well-being question, may have affected the answers given by the respondents. While we do not believe this distorted our data analysis, comparisons to studies with a different survey design must be made with caution.

The student sample comes with a few biases, unavoidable due to the academic environment of the survey. First, our respondents are mostly in their early twenties. People have the highest tendency for social comparisons in their late teens and early twenties (Buunk et al., 2020: 1; Callan et al., 2015: 196). Moreover, students are in a transitional phase at the beginning of their careers. Our results may, therefore, be biased toward upward comparisons, which are linked to the uncertainties of life transitions (Lockwood et al., 2012: 994). The competitive university environment may further encourage upward comparisons. Second, our student sample may have a middle-class bias. Due to greater social mobility, a middle-class context encourages social comparison (see, e.g., Steijn et al., 1998; Swencionis & Fiske, 2020: 258). Adding to this, women who make up the majority of our respondents are not only more inclined toward comparing themselves with others (Guimond & Chatard, 2014: 223) but may further bias our results towards upward comparisons (see, e.g., Pulford et al., (2018: 677) on female students). Third, Thailand is a collectivistic society, and people tend to compare themselves upward and more frequently. A similar bias may arise from the high-income inequality within Thailand (see above). In summary, we expect to find a tendency toward upward social comparisons in our data. Furthermore, online surveys are typically prone to self-selection and dropout issues. Those who dropped out may show a different social comparison pattern than those who continued the survey. It is also reasonable to assume that within the group of dropouts, a much higher share of non-comparer is hidden.

## Results

### Who are the True Reference Persons for Income Comparisons?

Table 1 shows the characteristics of the reference individuals based on the respondents’ subjective assessments. Respondents mostly compared their income to individuals who were better off, and two-thirds of all comparisons were made upwards. This is in accordance with the literature. Pronounced income inequality causes a higher incidence of upward social comparisons (Schneider, 2019: 411), and the high level of income inequality in Thailand might also explain the high share of upward comparers (Warr, 2007: 138). Moreover, collectivist societies such as Thailand tend to be less conducive to self-enhancement (Heine & Hamamura, 2007: 4; Kitayama et al., 1997: 1245).^13^ In line with this, people who score high on collectivism tend to make more upward and less downward comparisons (Chung & Mallery, 1999: 340; White & Lehman, 2005: 241). Other reasons that may encourage upward comparisons could be the competitive university environment and that students likely have low incomes but aim for jobs with high salaries. This may explain the low number of downward comparisons found in the sample. A quarter of the reference individuals have incomes similar to the respondents.

**Table 1:** Characteristics of respondents’ reference individuals for income comparison

Variables	Percentage of all named reference individuals (%)
*Income (n = 1200)*	
Lower	11
Similar	26
Higher	63
*Wealth (n = 1132)*	
Lower	9
Similar	37
Higher	55
*Social status (n = 1123)*	
Lower	8
Similar	50
Higher	43
*Role relationship (n = 1191)*	
Relative/partner	23
Friend	33
Acquaintance	9
Colleague/fellow student	6
Neighbour	2
Not known personally	23
Other	6
*Duration of relationship (n = 1199)*	
Less than 1 year	17
1 year to less than 4 years	36
4 years to less than 10 years	18
10 or more years	29
*Geographical distance (n = 1197)*	
Very close	28
Rather nearby	25
Rather far	37
I do not know	10
*Same sex (n = 1199)*	
Yes	65

248 respondents provided *n* = 1200 reference individuals; on average 4.8 reference individuals per respondent (29 respondents did not provide any reference individuals); different n-sizes are caused by missing values

Concerning the reference individuals’ wealth, we observe a similar picture. Very few reference individuals were less wealthy than respondents (9%), and most income comparisons were made with individuals perceived to be wealthier (55%). Around one-third of reference individuals had a similar estimated wealth to the respondents. Things are a little different when we look at the perceived social status of the reference individuals. Here, most income comparisons were made with individuals of the same or a similar social status (50%). However, respondents were more likely to compare themselves to individuals with a higher social status (43%) than with a lower perceived social status (8%).

The most important role relationship was friends, which made up one-third of all reference individuals (33%). Around one-fifth (23%) of reference individuals were relatives or partners. Thus, about half of all reference individuals were well known to the respondent. Interestingly, people who were not known personally to the respondent accounted for one-fifth of all reference individuals. Given the high amount of time respondents spend on social media every day, it was expected that influencers and celebrities would play an important role in our sample. This trend was also found in the qualitative interviews we conducted with students during the testing phase of our survey instrument. In contrast, the otherwise popular reference group, neighbours, only played a minor role in our student sample. Only 2% of all reference individuals were neighbours. However, it is noteworthy that over half of the reference individuals live in relatively close geographical proximity.

Data on the duration of the relationship shows that, on the one hand, reference individuals are subject to change over time (about 50% of reference individuals are known for four years or less). This finding is in accordance with the results of a study by Knight and Gunatilaka (2010: 113). They show that rural–urban work migrants in China changed their reference groups from co-villagers to city dwellers. On the other hand, there seems to be a core group of stable reference individuals: almost a third of the comparisons were made with individuals the respondents had known for more than ten years, usually family members.

### Comparison Patterns and their link to Subjective Well-Being

This section takes a closer look at the comparison patterns (Table 2). We look at the sample of respondents (n = 276) and show their comparison patterns aggregated from the positions of their reference individuals (see Sect. 3.2). Over half of the respondents (52%) are upward comparers. Our results also show that a relatively small proportion (11%) of respondents do not make income comparisons at all. This indicates that our strategy of using warm-up questions reduced the biases mentioned above.^14^

**Table 2:** Characterisation of comparison patterns

	Non-comparer (11%)	Upward comparer (52%)	Horizontal comparer (23%)	Fuzzy comparer (14%)	All
Subjective well-being (1–10)	8.1	7.0	7.6	7.6	7.3
*Big five personality traits Ø two items*					
Openness (1–7)	4.3	4.4	4.5	4.3	4.4
Conscientiousness (1–7)	3.7	4.1	4.2	4.0	4.1
Extraversion (1–7)	4.0	4.1	4.3	4.2	4.1
Agreeableness (1–7)	5.5	5.7	5.4	5.3	5.5
Neuroticism (1–7)	3.8	4.2	4.1	4.1	4.1
*SCO each orientation Ø of three items*					
Performance orientation (1–7)	3.0	3.7	3.7	3.5	3.6
Opinion orientation (1–7)	3.9	4.3	4.1	4.3	4.2
*Net income in Baht per month*					
Up to 2000	23%	15%	11%	18%	15%
2000–3999	19%	26%	29%	20%	25%
4000–5999	26%	20%	19%	28%	21%
More than 6000 Baht	32%	41%	41%	35%	39%
*Social media time in hours per day*					
Less than 3 h	23%	11%	11%	8%	12%
3 to less than 5 h	23%	29%	32%	35%	30%
5 to less than 7 h	23%	29%	37%	33%	31%
7 or more hours	32%	30%	21%	25%	27%
Female (1/0)	71%	83%	79%	80%	80%
Chiang Mai University (1/0)	29%	41%	38%	28%	37%
Age	20.4	20.4	20.5	20.3	20.4

n = 276; A detailed description of the measures behind the variables can be found in Table A 1 in the appendix

The different income comparison patterns cannot be linked to a personality type or other characteristics listed in Table 2. The only notable difference was in the social comparison orientation. As expected, the performance orientation of social comparisons (*How am I doing compared to others?*) is lower among non-comparers. We can only speculate, but this could be because these respondents see less value in social comparisons and, therefore, require less information regarding their standing compared to others. It is also interesting that the opinion orientation (*What do others think*/*How do others act in a similar situation to me?*) is similar across all comparison patterns. The opinion orientation is more action-oriented and less status-oriented and, thus, seems less relevant for comparing income.

Finally, on average, individuals in the group of non-comparers report the highest level of subjective well-being with 8.1 points on the 10-point scale. Horizontal and fuzzy comparers follow this with 7.6 points. Upward comparers report, on average, the lowest level of subjective well-being with 7.0 points. This is a first indication of a negative effect of upward income comparisons on subjective well-being.

In the following, we analyse the association between the comparison patterns and subjective well-being using regression models (see Sect. 0 for a detailed description of the variables). In doing so, we answer whether respondents are more satisfied when they compare their income upward instead of horizontally or not at all. Table 3 shows the OLS regression model results. We detected no problems with multicollinearity according to the variance inflation factor (VIF). Moreover, our relatively low R-square indicates that we do not have strong multicollinearity.^15^ According to the Breusch-Pagan test, heteroscedasticity is present. We use robust standard errors as they tend to provide more accurate measures of the true standard errors of a regression coefficient and are also suited to the problem of heteroskedasticity (Wooldridge, 2013: 276). Moreover, scatter and stem-and-leaf plots and leverage analytics did not show strong outliers in the data. We checked for non-linearity of the variables and specification errors and found none.^16^

**Table 3:** OLS regression: Is subjective well-being affected by income comparisons?

	Model 1				Model 2			
	Omega-squared*	Coefficients	Robust std. errors	*p* value	Omega-squared*	Coefficients	Robust std. errors	*p* value
Comparison pattern	**0.014**				**0.010**			
Upward	Reference: non-comparer	− 0.800	0.360	0.027	Reference: upward comparer	–	–	–
Horizontal		− 0.256	0.399	0.521		0.572	0.288	0.048
Fuzzy		− 0.465	0.416	0.265		0.391	0.332	0.241
*Big Five personality traits Ø two items*								
Openness (1–7)	−0.002	0.079	0.117	0.501	− 0.004	0.036	0.128	0.781
Conscientiousness (1–7)	0.003	0.131	0.105	0.215	− 0.002	0.086	0.116	0.458
Extraversion (1–7)	− 0.003	0.058	0.113	0.610	− 0.004	0.011	0.123	0.931
Agreeableness (1–7)	**0.021**	0.290	0.111	0.009	**0.032**	0.370	0.129	0.005
Neuroticism (1–7)	**0.034**	− 0.331	0.108	0.002	**0.038**	− 0.360	0.124	0.004
*SCO each orientation Ø of three items*								
Performance orientation (1–7)	**0.019**	− 0.239	0.092	0.010	0.008	− 0.180	0.098	0.069
Opinion orientation (1–7)	**0.023**	0.217	0.085	0.011	**0.012**	0.173	0.091	0.057
*Net income in Baht per month*	**0.013**				0.005			
2000–3999	Reference: up to 2000	− 0.075	0.406	0.854	Reference: up to 2000	0.019	0.430	0.965
4000–5999		0.498	0.409	0.224		0.298	0.455	0.513
More than 6000		0.732	0.411	0.076		0.687	0.449	0.128
*Social media time in hours per day*	**0.013**				**0.011**			
3 to less than 5 h	Reference: less than 3 h	− 0.653	0.405	0.108	Reference: less than 3 h	− 0.700	0.460	0.129
5 to less than 7 h		− 0.475	0.397	0.232		− 0.602	0.464	0.195
7 or more hours		− 0.997	0.390	0.011		− 1.042	0.458	0.024
Female: (1/0)	− 0.004	− 0.007	0.317	0.983	− 0.004	− 0.043	0.344	0.900
Chiang Mai University: (1/0)	**0.076**	− 1.346	0.295	0.000	**0.068**	− 1.300	0.324	0.000
Age	0.000	− 0.095	0.089	0.285	0.001	− 0.105	0.098	0.286
Constant	–	9.107	2.226	0.000	–	8.823	2.601	0.001
N	276				245			
R-squared	0.257				0.254			

*Based on OLS regression results with normal standard errors (results are not shown here)

We calculated omega-squared as a measure of effect size.^17^ We discuss only variables showing an omega-squared of at least 0.01, which is considered a small effect. Everything below that threshold is regarded as negligible. Such a negligible association is, for example, the association of subjective well-being with being a female respondent. The associations between subjective well-being and the covariates are relatively small, but this is typical for variables explaining subjective well-being.

All income comparison patterns, except for non-comparers, are negatively related to the level of subjective well-being (see Table 3, model 1). This means that respondents who said they did not compare their incomes are predicted to report higher levels of subjective well-being. As pointed out by Alderson and Katz-Gerro (2016: 37) and Clark et al., (2013: 16), the more important it is for individuals to compare themselves to others, the less satisfied they are with their lives.

Among those who compare their income to others, upward comparers are least satisfied with their lives. They have the largest negative coefficient among the comparison patterns. In model 2, we run a regression without the non-comparers. This model underlines once more the negative implication of comparing upwards. For those who compare at all, horizontal and fuzzy comparisons are more favourable than upward comparisons, which are negatively linked with subjective well-being. Thus, negative emotions such as envy may outweigh the positive effects of upward income comparisons (Clark & Senik, 2010: 591). Usually, in low-income countries, the signal effect dominates over the envy effect, and in high-income countries, it is the other way round (Brockmann & Yan, 2013: 142–143). Our results underline that a student sample in an upper-middle-income country like Thailand is comparable to developed countries. Leites and Ramos (2022: 21) confirm for another middle-income country (Uruguay) that envy dominates the signalling effect.

Collectivistic societies, such as Thailand, often display low social/occupational mobility (Hofstede, 2001: 118, 244 ff.; Hofstede et al., 2005: 119). Therefore, upward income comparisons may not result in a signalling effect or be viewed as information for self-improvement. Instead, looking upward causes frustration if the respondents do not see the possibility of future social advancement. The positive sign of the horizontal comparer pattern indicates conforming and reassuring effects.

The SCO scale measures the intensity of social comparisons made by a respondent (see Sect. 0). The performance orientation of social comparisons (*How am I doing compared to others?*) is negatively related to subjective well-being, as expected (Gibbons & Buunk, 1999: 133; Schneider & Schupp, 2014: 782). Contrary to the literature, we found a positive association between subjective well-being and opinion orientation (*What do others think*/*How do others act in a similar situation to me?*). A high score on the opinion orientation is associated with, on average, higher levels of subjective well-being. This may also indicate a more forward-looking person who compares with others to gain information to improve or confirm their own decisions.

Two out of five personality traits had a sizeable effect, according to omega-squared. Agreeableness can facilitate positive experiences, which may increase subjective well-being (Hayes & Joseph, 2003: 723, 726). Thus, not surprisingly, the variable agreeableness shows a positive relationship with subjective well-being. In line with other research, neuroticism—a personality trait characterised by worrying, depression, and anxiety (Buunk & Gibbons, 2007: 12)—is negatively associated with subjective well-being (Anglim et al., 2020: 279).

Neuroticism is deemed especially relevant for social comparisons, as individuals with high levels of neuroticism have a greater need for comparison and a tendency to compare upwards and report lower subjective well-being after making social comparisons (VanderZee et al., 1996: 551). We, therefore, added an interaction term to our regression models to check whether neuroticism affects the relationship between comparison patterns and subjective well-being (see Table 4). Model 1.2 shows the interaction with neuroticism as a continuous variable. For the sake of easier interpretation, the neuroticism variable in model 1.3 was, however, introduced as a dummy variable. The dummy variable equals one (1) if the value for neuroticism is 3.5 or higher, indicating a respondent with high levels of neuroticism, and it equals zero (0) if the value is lower than 3.5.^18^ The coefficient of the interaction term between neuroticism and comparison patterns shows that individuals who are high on the neuroticism scale and compare horizontally or upwards are associated with lower subjective well-being (model 1.3) compared to non-comparers. High neuroticism and comparing horizontally may cause a negative signal effect. This means that respondents could be worried that their income may not rise or even fall in the future. Being high on the neuroticism scale and comparing upwards may trigger envy or shame towards those who earn more. However, being low on the neuroticism scale and comparing horizontally is associated with even higher subjective well-being than non-comparing respondents. This may be related to affirmation of themselves and their decisions.

**Table 4:** OLS regression: Is subjective well-being affected by income comparisons? Interaction effects model

	Model 1.2				Model 1.3			
	Omega-squared*	Coefficients	Robust std. errors	p value	Omega-squared*	Coefficients	Robust std. errors	p value
Comparison pattern	**0.006**				**0.007**			
upward	Reference: non-comparer	0.899	1.046	0.391	Reference: non-comparer	0.459	0.597	0.443
horizontal		0.879	1.026	0.393		1.256	0.672	0.063
fuzzy		− 1.672	1.164	0.152		− 0.334	0.739	0.652
Comparison pattern/neuroticism**	**0.013**				**0.020**			
upward # neuroticism		− 0.430	0.245	0.080		− 1.794	0.735	0.015
horizontal # neuroticism		− 0.295	0.242	0.224		− 2.006	0.839	0.017
fuzzy # neuroticism		0.306	0.291	0.294		− 0.227	0.898	0.800
*Big Five personality traits Ø two items*								
Openness (1–7)	− 0.002	0.068	0.119	0.570	− 0.004	0.031	0.118	0.791
Conscientiousness (1–7)	0.002	0.124	0.101	0.224	0.000	0.105	0.103	0.311
Extraversion (1–7)	− 0.003	0.051	0.112	0.653	− 0.003	0.058	0.115	0.614
Agreeableness (1–7)	**0.024**	0.302	0.109	0.006	**0.011**	0.222	0.109	0.042
Neuroticism (1–7)**	**0.009**	− 0.103	0.185	0.581	**0.012**	0.353	0.649	0.587
*SCO each orientation Ø of three items*								
Performance orientation *(1–7)*	**0.015**	− 0.215	0.092	0.020	**0.016**	− 0.219	0.093	0.020
Opinion orientation *(1–7)*	**0.027**	0.235	0.083	0.005	**0.020**	0.201	0.082	0.015
*Net income in Baht per month*	0.005				0.006			
2000–3999	Reference: up to 2000	− 0.189	0.414	0.648	Reference: up to 2000	0.015	0.409	0.971
4000–5999		0.326	0.422	0.441		0.434	0.420	0.302
More than 6000		0.508	0.430	0.239		0.670	0.416	0.109
*Social media time in hours per day*	**0.014**				**0.018**			
3 to less than 5 h	reference: less than 3 h	− 0.593	0.401	0.141	Reference: less than 3 h	− 0.653	0.400	0.104
5 to less than 7 h		− 0.426	0.395	0.281		− 0.595	0.401	0.139
7 or more hours		− 0.961	0.386	0.013		− 1.078	0.390	0.006
Female: (1/0)	− 0.004	− 0.038	0.314	0.905	− 0.004	− 0.040	0.297	0.894
Chiang Mai University: (1/0)	**0.068**	− 1.273	0.295	0.000	**0.075**	− 1.317	0.290	0.00
Age	0.002	− 0.111	0.087	0.203	0.006	− 0.146	0.082	0.076
Constant	−	8.572	2.101	0.000	–	9.394	2.088	0.000
N	276				276			
R-squared	0.278				0.286			

*Based on OLS regression results with normal standard errors (results are not shown here)

**In Model 1.2 the variable is continuous. In Model 1.3, we used a dummy which turns 1 for high neuroticism [for values ≥ 3.5]

## Conclusions

We developed and implemented a new survey instrument to identify the reference individuals our respondents use to compare their incomes. This allowed us to investigate the composition of income reference groups in more detail. We found that reference individuals come from a range of social groups. Although this finding was expected, it is rarely documented in the literature and highlights the risk of over-simplification when using an a priori defined reference group. Furthermore, surveys that apply selection methods to identify reference groups should provide a broader range of role relationships that include, for example, social media influencers or celebrities, as these made up the third most important group in our sample. Our survey instrument also allowed us to conduct a detailed investigation of the direction of income comparisons. In contrast to the literature on general social comparisons, only a few respondents in our sample reported downward income comparisons. Instead, most comparisons were upward. Additionally, we found that horizontal income comparisons frequently occurred, despite being largely overlooked in the literature.

We identified four main income comparison patterns: upward, horizontal, fuzzy, and non-comparers. These patterns did not show much variation regarding socio-economic characteristics and personality traits. However, non-comparers had the highest level of subjective well-being, while upward comparers had the lowest. Our regression model confirmed this strongly negative relationship. What is more, we show that income comparisons per se seem to be negatively linked to the level of subjective well-being but to a different degree. Among those who compare their income to others, horizontal comparers are most satisfied with their lives. This may be because comparisons with people of a similar income level have a confirming and reassuring effect. Upward comparers are least satisfied, implying that the positive effects of upward comparisons, such as the tunnel effect, might be outweighed by the negative effects caused by emotions such as envy.

Although personality traits did not appear to make much of a difference in the choice of comparison patterns, they played a role in the level of subjective well-being. In line with the literature, we found that agreeableness is positively associated with subjective well-being and neuroticism is negatively associated with it. We further identified an interesting interaction between high levels of neuroticism and comparison patterns. The interaction strengthens the negative association with subjective well-being for upward and horizontal comparisons. Low levels of neuroticism coupled with horizontal comparisons result in the highest level of subjective well-being. These findings highlight the importance of collecting information on personality traits in subjective well-being research.

Our results must be interpreted with some limitations in mind (discussed in detail in Sect. 3.4). Our empirical study is exploratory and refers to a convenience student sample in the specific context of a collectivistic society. Thus, the measurement instrument of individualised reference groups should be validated with surveys covering a broader range of population segments and culturally diverse societies. Notwithstanding these limitations in this first empirical application, our novel approach to identifying reference individuals has proven straightforward and feasible. Questions about self-reported income comparisons and reference individuals can thus be a valuable addition to current survey instruments. They should not (yet) fully replace standard measures of reference groups because comparisons with abstract reference groups, such as fellow citizens, and personalised comparisons with true reference persons are not mutually exclusive; they may fulfil different informational needs and thus should be distinguished (Locke, 2007: 224; Marsh et al., 2008: 519). Our research is an important starting point for bringing more clarity to this debate.

## Appendix

See Table

**Table 5:** Summary statistics of variables used in the regression

Variables	Mean	Std. dev	1 (%)	2 (%)	3 (%)	4 (%)	5 (%)	6 (%)	7 (%)	8	9	10
*Social comparison scale**												
*Performance—*I always pay a lot of attention to how I do things compared with how others do things	3.5	1.8	17	13	22	18	17	7	5	–	–	–
*Performance—*I often compare how I am doing socially (e.g. social skills, popularity) with other people	3.3	1.7	17	18	21	19	15	6	4	–	–	––
*Performance—*I am the type of person to compare myself often with others. (**reverse, recoded**)	4.0	2.0	17	12	10	14	19	11	16	–	–	–
O*pinion*—I often try to find out what others facing similar problems think	3.9	1.8	12	13	17	20	20	11	8	–	–	–
O*pinion*—I always like to know what others would do in a similar situation	4.3	1.8	8	10	13	23	21	16	9	–	–	–
O*pinion*—If I want to learn more about something, I try to find out what others think about it	4.5	1.7	4	12	12	24	20	17	12	–	–	–
*Big Five personality traits*—I see myself as someone who …*												
*Openness—*… has an active imagination	4.9	1.5	3	3	10	21	26	21	17	–	–	–
*Openness—*… has (**many**) artistic interests. (**reverse, recoded**)	3.9	1.8	15	9	20	21	14	9	12	–	–	–
*Conscientiousness—*… does a thorough job	4.5	1.4	2	6	13	31	24	9	15	–	–	–
*Conscientiousness—*… tends (**not**) to be lazy. (**reverse, recoded**)	3.7	1.6	11	14	21	24	16	4	11	–	–	–
*Extraversion—*… is outgoing, sociable	4.9	1.6	2	5	11	22	24	21	16	–	–	–
*Extraversion—*… is (**not**) reserved. (**reverse, recoded**)	3.3	1.5	17	10	26	26	14	3	3	–	–	–
A*greeableness—*… is generally trusting	6.0	1.2	0	0	3	10	16	44	26	–	–	–
A*greeableness—*… tends (**not**) to find fault with others. (**reverse, recoded**)	5.1	1.7	4	5	11	15	15	26	24	–	––	–
*Neuroticism—*… gets nervous easily	4.9	1.7	3	4	10	22	26	21	14	–	–	–
*Neuroticism—*… is (**not**) relaxed, handles stress well. (**reverse, recoded**)	3.3	1.4	13	17	24	27	12	1	5	–	–	–
Subjective well-being—All things considered, how satisfied are you with your life?	7.3	2.1	0	0	4	5	12	13	20	13%	13%	20%

n = 276; to keep interviews short, we used simplified (but proven) measurements for the measurement of subjective well-being, the social comparison scale, and the Big Five personality traits.

*1 does not apply at all—7 applies completely

**5**

## Footnotes

1 In this paper, we follow Veenhoven (1984: 23) and understand subjective well-being as `the degree to which an individual judges the overall quality of his life-as-a-whole favourably'. We make use of self-reported life satisfaction as an indicator of subjective well-being.

2 Research on inequality has shown that people systematically fail to locate their income position within society correctly. People are biased towards the mean. Better-off people tend to overestimate, and poorer people tend to underestimate the income of others (Engelhardt & Wagener, 2018: 744; Gimpelson & Treisman, 2015: 1). It is reasonable to assume that they are likewise not aware of the average income of, for instance, an age cohort.

3 This seems to be especially relevant for comparisons via social media. The constant use of mobile internet devices (mostly smartphones) exposes users to a permanent stream of idealised images from social network sites, which can also be seen as upward comparisons and may result in negative emotions such as envy or depression (Schmuck et al., 2019: 2, 7).

4 In social psychology, Wheeler and Miyake (1992) developed a method called the Rochester Social Comparison Record, which asks respondents to complete diary records of their everyday social comparisons. However, diary methods risk missing important reference persons absent during the diary period (usually one to two weeks).

5 This can change depending on the cultural context;in Japan, friends are the most important reference group (Yamada & Sato, 2013: 40).

6 Individuals in peer networks tend to be especially homogeneous because of the principle of homophily, the selection of friends/partners from similar backgrounds (McPherson et al., 2001: 415).

7 Qualitative interviews before the survey revealed that respondents had no problem to identify real individuals as their reference points. Our wording of 'real' people excludes fictional characters. While this potentially limits the reference choice of the respondents, our qualitative research did not suggest that this limitation may be a problem.

8 The social desirability bias is likely less relevant in our case, as the interviews were self-administered, and, thus, respondents did not desire to please the enumerator.

9 We asked the subjective well-being question almost at the end of the survey.

10 They also state that fixed-effects models should be used to account for time-consistent unobserved individual heterogeneity in panel settings. Since we do not have panel data, we include time-consistent variables such as Big Five personality traits and a measure of social comparison orientation (SCO) that otherwise cannot be considered in fixed-effects models.

11 The literature identifies health as a robust determinant of subjective well-being (see e.g. Brockmann & Delhey, 2010: 2; Dolan et al., 2008: 100; Knight & Gunatilaka, 2010: 117). Our subjective health status variableis not included in our model. It was asked in a grid format together with the subjective well-being question (measured with the same scale). This resulted in a strong order effect, which "seizes" the model when we add the health variable into the regression model. We could reduce the effect by rescaling the subjective health variable to a dummy. However, we decided not to include this variable due to the identified issues.

12 In accordance with Jenkins and Quintana-Ascencio (2020: 11) this N suffices the requirements for regression analysis in observational studies.

13 However, other researchers state that self-enhancement is culturally universal (O’Mara et al., 2012: 157; Sedikides et al., 2005: 539).

14 Studies using global self-reports on income reference groups show varying levels of non-comparing respondents, e.g. Burchell and Yagil (1997: 741) 52%, Clark and Senik (2010: 576) 36%, Yamada and Sato (2013: 40) 25%, Knight et al., (2009: 638) 14%, and Alderson and Katz-Gerro (2016: 36) 13%.

15 According to Wooldridge (2013: 39), low R-squared values of regression models are quite common in the social sciences. Moreover, even if we cannot observe and include all determinants of subjective well-being in our model, this does not mean that our OLS estimation has no explanatory worth. The regression equation may well be a good estimator of the *ceteris paribus* relationship between subjective well-being and comparison patterns. Compared to other works in the field, our R-square is within the usual ranges: in most regressions on subjective well-being only a small part of the variance is explained by observable characteristics (see e.g. Senik, 2005: 46).

16 Having only one reference individual automatically creates an income comparison pattern of upward or horizontal comparer. We added a dummy variable for having only one reference individual in our model to control for this. The effect size of this variable was very small, and it did not change the results. But it lowered the Akaike-Information-Criterion (AIC), and Bayesian-Information-Criterion (BIC). Therefore, we did not include it in our models. We further ruled out a bias towards strong ties created by the name generator. Individuals who are close to the respondent may be over-reported as they are the first to come to mind (results are available upon request).

17 Omega-squared measures the extent to which the total variance of a dependent metric variable (e.g. subjective well-being) is explained by an independent variable (e.g. gender). We refrain from interpreting the p-values and standard errors but still provide them to interested readers. The reason for this is that we do not draw inductive inferences beyond the limits of our student sample to a specific population, as we did not apply a random process of data generation that can be hypothetically replicated (Hirschauer et al., 2020: 72).

18 Despite this loss of information, the regression diagnostics in R-squared, AIC, and BIC improved.

## References

[CR1] Akay A, Bargain O, Zimmermann KF (2012). Relative concerns of rural-to-urban migrants in China. Journal of Economic Behavior & Organization.

[CR2] Alderson AS, Katz-Gerro T (2016). Compared to whom? Inequality, social comparison, and happiness in the United States. Social Forces.

[CR3] Anglim J, Horwood S, Smillie LD, Marrero RJ, Wood JK (2020). Predicting psychological and subjective well-being from personality: A meta-analysis. Psychological Bulletin.

[CR4] Asadullah MN, Chaudhury N (2012). Subjective well-being and relative poverty in rural Bangladesh. Journal of Economic Psychology.

[CR5] Becchetti L, Castriota S, Corrado L, Ricca EG (2013). Beyond the Joneses: Inter-country income comparisons and happiness. The Journal of Socio-Economics.

[CR6] Begley TM, Tan W-L (2001). The socio-cultural environment for entrepreneurship: A comparison between East Asian and Anglo-Saxon countries. Journal of International Business Studies.

[CR7] Bellani L (2012). Multidimensional indexes of deprivation: The introduction of reference groups weights. Journal of Economic Inequality.

[CR8] Boyce CJ, Wood AM, Powdthavee N (2013). Is personality fixed? Personality changes as much as “variable” economic factors and more strongly predicts changes to life satisfaction. Social Indicators Research.

[CR9] Brockmann H, Delhey J (2010). Introduction: The dynamics of happiness and the dynamics of happiness research. Social Indicators Research.

[CR10] Brockmann H, Yan S, Brockmann K, Delhey J (2013). My car is bigger than yours: Consumption, status competition, and happiness in times of affluence. Human happiness and the pursuit of maximization.

[CR11] Brown S, Gray D, Roberts J (2015). The relative income hypothesis: A comparison of methods. Economics Letters.

[CR12] Bruchmann K, Evans AT (2013). Abstract mind-sets and social comparison: When global comparisons matter. Social Psychological and Personality Science.

[CR13] Burchell B, Yagil D (1997). Socioeconomic and political initiators of pay comparison. Work, Employment and Society.

[CR14] Buunk AP, Dijkstra PD, Bosma HA (2020). Changes in social comparison orientation over the life-span. Journal of Clinical & Developmental Psychology.

[CR15] Buunk AP, Gibbons FX, Guimond S (2006). Social comparison orientation: A new perspective on those who do and those who don't compare with others. Social comparison and social psychology: Understanding cognition, intergroup relations, and culture.

[CR16] Buunk AP, Gibbons FX (2007). Social comparison: The end of a theory and the emergence of a field. Organizational Behavior and Human Decision Processes.

[CR17] Callan MJ, Kim H, Matthews WJ (2015). Age differences in social comparison tendency and personal relative deprivation. Personality and Individual Differences.

[CR18] Chung T, Mallery P (1999). Social comparison, individualism-collectivism, and self-esteem in China and the United States. Current Psychology.

[CR19] Clark, A. E., Senik, C., & Yamada, K. (2013). The Joneses in Japan: Income comparisons and financial satisfaction. In *Discussion paper*. Osaka: The Institute of Social and Economic Research, Osaka University.

[CR20] Clark AE, Frijters P, Shields MA (2008). Relative income, happiness, and utility: An explanation for the Easterlin paradox and other puzzles. Journal of Economic Literature.

[CR21] Clark AE, Masclet D, Villeval MC (2010). Effort and comparison income: Experimental and survey evidence. Industrial and Labor Relations Review.

[CR22] Clark AE, Senik C (2010). Who compares to whom? The anatomy of income comparisons in Europe. The Economic Journal.

[CR23] Clark AE, Westergard-Nielsen N, Kristensen N (2009). Economic satisfaction and income rank in small neighbourhoods. Journal of the European Economic Association.

[CR24] Corcoran K, Mussweiler T (2009). The efficiency of social comparisons with routine standards. Social Cognition.

[CR25] Diener E (1984). Subjective wellbeing. Psychological Bulletin.

[CR26] Diener E, Emmons RA, Larsen RJ, Griffin S (1985). The satisfaction with life scale. Journal of Personality Assessment.

[CR27] Diener E, Fujita F, Buunk BP, Gibbons FX (1997). Social comparisons and subjective well-being. Health, coping, and well-being: Perspectives from social comparison theory.

[CR28] Dolan P, Peasgood T, White M (2008). Do we really know what makes us happy? A review of the economic literature on the factors associated with subjective well-being. Journal of Economic Psychology.

[CR29] Draper J, Selway JS (2019). A new dataset on horizontal structural ethnic inequalities in Thailand in order to address Sustainable Development Goal 10. Social Indicators Research.

[CR30] Dufhues T, Buchenrieder G, Euler DG, Mungkung N (2011). Network based social capital and individual loan repayment performance. Journal of Development Studies.

[CR31] Easterlin RA, David PA, Reder MW (1974). Does economic growth improve the human lot?. Nations and households in economic growth: Essays in honour of Moses Abramovitz.

[CR32] Easterlin RA (1995). Will raising the incomes of all increase the happiness of all?. Journal of Economic Behavior & Organization.

[CR33] Engelhardt C, Wagener A (2018). What do Germans think and know about income inequality? A survey experiment. Socio-Economic Review.

[CR34] Falk A, Knell M (2004). Choosing the Joneses: Endogenous goals and reference standards. The Scandinavian Journal of Economics.

[CR35] Ferrer-i-Carbonell A (2005). Income and well-being: An empirical analysis of the comparison income effect. Journal of Public Economics.

[CR36] Ferrer-i-Carbonell A, Frijters P (2004). How important is methodology for the estimates of the determinants of happiness?. The Economic Journal.

[CR37] Festinger L (1954). A theory of social comparison processes. Human Relations.

[CR38] Fujita F, Eid M, Larsen RJ (2008). The frequency of social comparison and its relation to subjective well-being. The science of subjective well-being.

[CR39] Gerber J, Wheeler L, Suls J (2018). A social comparison theory meta-analysis 60+ years on. Psychological Bulletin.

[CR40] Gibbons FX, Buunk AP (1999). Individual differences in social comparison: Development of a scale of social comparison orientation. Journal of Personality and Social Psychology.

[CR41] Gimpelson, V., & Treisman, D. (2015). Misperceiving inequality. In *Working paper* (vol. 21174). Cambridge: National Bureau of Economic Research.

[CR42] Goerke L, Pannenberg M (2015). Direct evidence for income comparisons and subjective well-being across reference groups. Economics Letters.

[CR43] Goldberg LR (1993). The structure of phenotypic personality traits. American Psychologist.

[CR44] Gugushvili A (2020). Which socio-economic comparison groups do individuals choose and why?. European Societies.

[CR45] Guimond S, Chatard A, Križan Z, Gibbons FX (2014). Basic principles of social comparison: Does gender matter. Communal functions of social comparison.

[CR46] Hayes N, Joseph S (2003). Big 5 correlates of three measures of subjective well-being. Personality and Individual Differences.

[CR47] Heine SJ, Hamamura T (2007). In search of East Asian self-enhancement. Personality and Social Psychology Review.

[CR48] Hemphill KJ, Lehman DR (1991). Social comparisons and their affective consequences: The importance of comparison dimension and individual difference variables. Journal of Social and Clinical Psychology.

[CR49] Hirschauer N, Grüner S, Mußhoff O, Becker C, Jantsch A (2020). Can p-values be meaningfully interpreted without random sampling?. Statistics Surveys.

[CR50] Hirschman AO, Rothschild M (1973). The changing tolerance for income inequality in the course of economic development. The Quarterly Journal of Economics.

[CR51] Hofstede GH (2001). Culture's consequences: Comparing values, behaviors, institutions, and organisations across nations.

[CR52] Hofstede GH, Hofstede GJ, Minkov M (2005). Cultures and organizations: Software of the mind.

[CR53] Hopkins E (2008). Inequality, happiness and relative concerns: What actually is their relationship?. Journal of Economic Inequality.

[CR54] Hyll W, Schneider L (2014). Relative deprivation and migration preferences. Economics Letters.

[CR55] Hyman HH, Singer E, Hyman HH, Singer E (1968). Introduction. Readings in reference group theory and research.

[CR56] Inglehart R, Foa R, Peterson C, Welzel C (2008). Development, freedom, and rising happiness: A global perspective (1981–2007). Perspectives on Psychological Science.

[CR57] Jantsch, A., Le Blanc, J., & Schmidt, T. (2022). Wealth and subjective well-being in Germany. In *Discussion paper* (vol. 11). Frankfurt: Deutsche Bundesbank.

[CR58] Jenkins DG, Quintana-Ascencio PF (2020). A solution to minimum sample size for regressions. PLoS ONE.

[CR59] Kim H, Callan MJ, Gheorghiu AI, Skylark WJ (2018). Social comparison processes in the experience of personal relative deprivation. Journal of Applied Social Psychology.

[CR60] Kingdon GG, Knight J (2007). Community, comparisons and subjective well-being in a divided society. Journal of Economic Behavior & Organization.

[CR61] Kitayama S, Markus HR, Matsumoto H, Norasakkunkit V (1997). Individual and collective processes in the construction of the self: Self-enhancement in the United States and self-criticism in Japan. Journal of Personality and Social Psychology.

[CR62] Knight J, Gunatilaka R (2010). Great expectations? The subjective well-being of rural-urban migrants in China. World Development.

[CR63] Knight J, Song L, Gunatilaka R (2009). Subjective well-being and its determinants in rural China. China Economic Review.

[CR64] Lang M, Reynolds RA, Woods R, Baker JD (2007). Dual-mode electronic survey lessons and experiences. Handbook of research on electronic surveys and measurements.

[CR65] Laovakul D, Pasuk P, Baker C, Pasuk P, Baker C (2016). Concentration of land and other wealth in Thailand. Unequal Thailand: Aspects of income, wealth and power.

[CR66] Leites M, Ramos X (2022). The effect of relative income concerns on life satisfaction: Relative deprivation and loss aversion. Journal of Happiness Studies.

[CR67] Locke KD (2007). Personalized and generalized comparisons: Causes and consequences of variations in the focus of social comparisons. Personality Social Psychology Bulletin.

[CR68] Lockwood P (2002). Could it happen to you? Predicting the impact of downward comparisons on the self. Journal of Personality Social Psychology.

[CR69] Lockwood P, Shaughnessy SC, Fortune JL, Tong M-O (2012). Social comparisons in novel situations: Finding inspiration during life transitions. Personality and Social Psychology Bulletin.

[CR70] Major B, Testa M, Blysma WH, Suls J, Wills TA (1991). Responses to upward and downward social comparisons: The impact of esteem-relevance and perceived control. Social comparison - Contemporary theory and research.

[CR71] Marsden PV (1990). Network data and measurement. Annual Review of Sociology.

[CR72] Marsh HW, Trautwein U, Lüdtke O, Köller O (2008). Social comparison and big-fish-little-pond effects on self-concept and other self-belief constructs: Role of generalized and specific others. Journal of Educational Psychology.

[CR73] McBride M (2010). Money, happiness, and aspirations: An experimental study. Journal of Economic Behavior & Organization.

[CR74] McCallister L, Fischer CS (1978). A procedure for surveying personal networks. Sociological Methods and Research.

[CR75] McPherson M, Smith-Lovin L, Cook JM (2001). Birds of a feather: Homophily in social networks. Annual Review of Sociology.

[CR76] Merton RK (1995). The Thomas theorem and the Matthews effect. Social Forces.

[CR77] Mussweiler T, Rüter K (2003). What friends are for! The use of routine standards in social comparison. Journal of Personality and Social Psychology.

[CR78] O’Mara EM, Gaertner L, Sedikides C, Zhou X, Liu Y (2012). A longitudinal-experimental test of the panculturality of self-enhancement: Self-enhancement promotes psychological well-being both in the west and the east. Journal of Research in Personality.

[CR79] Olivos F, Olivos-Jara P, Browne M (2021). Asymmetric social comparison and life satisfaction in social networks. Journal of Happiness Studies.

[CR80] Pasuk P (2016). Inequality, wealth and Thailand’s politics. Journal of Contemporary Asia.

[CR81] Perez-Asenjo E (2011). If happiness is relative, against whom do we compare ourselves? Implications for labour supply. Journal of Population Economics.

[CR82] Powdthavee N (2009). How important is rank to individual perception of economic standing? A within-community analysis. Journal of Economic Inequality.

[CR83] Pulford BD, Woodward B, Taylor E (2018). Do social comparisons in academic settings relate to gender and academic self-confidence?. Social Psychology of Education.

[CR84] Rammstedt B, John OP (2007). Measuring personality in one minute or less: A 10-item short version of the Big Five Inventory in English and German. Journal of Research in Personality.

[CR85] Runciman WG (1966). Relative deprivation and social justice: A study of attitudes to social inequality in twentieth-century England.

[CR86] Schmuck D, Karsay K, Matthes J, Stevic A (2019). “Looking Up and Feeling Down” the influence of mobile social networking site use on upward social comparison, self-esteem, and well-being of adult smartphone users. Telematics and Informatics.

[CR87] Schneider SM (2019). Why income inequality Is dissatisfying—perceptions of social status and the inequality-satisfaction link in Europe. European Sociological Review.

[CR88] Schneider SM, Schupp J (2014). Individual differences in social comparison and its consequences for life satisfaction: Introducing a short scale of the Iowa-Netherlands comparison orientation measure. Social Indicators Research.

[CR89] Sedikides C, Gaertner L, Vevea JL (2005). Pancultural self-enhancement reloaded: A meta-analytic reply to Heine (2005). Journal of Personality and Social Psychology.

[CR90] Senik C (2004). When information dominates comparison: Learning from Russian subjective panel data. Journal of Public Economics.

[CR91] Senik C (2005). Income distribution and well–being: What can we learn from subjective data?. Journal of Economic Surveys.

[CR92] Senik C (2009). Direct evidence on income comparisons and their welfare effects. Journal of Economic Behavior & Organization.

[CR93] Smith RH, Suls JM, Wheeler L (2000). Assimilative and contrastive emotional reactions to upward and downward social comparisons. Handbook of social comparison.

[CR94] Snijders TAB (1999). Prologue to the measurement of social capital. The Tocqueville Review.

[CR95] Steijn B, Berting J, De Jong M-J (1998). Economic restructuring and the growing uncertainty of the middle class.

[CR96] Swencionis JK, Fiske ST, Suls J, Collins RL, Wheeler L (2020). Stereotypes and relative social status in social comparisons. Social comparison, judgment, and behavior.

[CR97] Taylor SE, Neter E, Wayment HA (1995). Self-evaluation processes. Personality and Social Psychology Bulletin.

[CR98] Taylor SE, Wood JV, Lichtman RR (1983). It could be worse: Selective evaluation as a response to victimization. Journal of Social Issues.

[CR99] van Praag B (2011). Well-being inequality and reference groups: An agenda for new research. Journal of Economic Inequality.

[CR100] VanderZee K, Buunk B, Sanderman R (1996). The relationship between social comparison processes and personality. Personality and Individual Differences.

[CR101] Veenhoven R (1984). Conditions of happiness.

[CR102] Warr P (2007). Long-term economic performance in Thailand. ASEAN Economic Bulletin.

[CR103] Wheeler L, Miyake K (1992). Social comparison in everyday life. Journal of Personality and Social Psychology.

[CR104] White K, Lehman DR (2005). Culture and social comparison seeking: The role of self-motives. Personality and Social Psychology Bulletin.

[CR105] Wills TA (1981). Downward comparison principles in social psychology. Psychological Bulletin.

[CR106] Wolbring T, Keuschnigg M, Negele E (2013). Needs, comparisons, and adaptation: The importance of relative income for life satisfaction. European Sociological Review.

[CR107] Wood JV, Taylor KL, Suls J, Wills TA (1991). Serving self-relevant goals through social comparison. Social comparison: Contemporary theory and research.

[CR108] Wooldridge JM (2013). Introductory econometrics: A modern approach.

[CR109] World Bank (2021). World development indicators.

[CR110] Wu, H. F. (2020). Relative income status within marriage and subjective well-being in China: Evidence from observational and quasi-experimental data. *Journal of Happiness Studies*, 1–20.

[CR111] Yamada K, Sato M (2013). Another avenue for anatomy of income comparisons: Evidence from hypothetical choice experiments. Journal of Economic Behavior and Organization.

[CR112] Zell E, Alicke MD (2010). The local dominance effect in self-evaluation: Evidence and explanations. Personality Social Psychology Review.

